# Physiopathology of Osteoporosis: Nursing Involvement and Management

**DOI:** 10.3390/biomedicines11041220

**Published:** 2023-04-19

**Authors:** Sandra Valenzuela-Martínez, María Jesús Ramírez-Expósito, María Pilar Carrera-González, José Manuel Martínez-Martos

**Affiliations:** 1 Experimental and Clinical Physiopathology Research Group CTS-1039, Department of Health Sciences, Faculty of Health Sciences, University of Jaén, Campus Universitario Las Lagunillas, 23009 Jaén, Spain; 2 Department of Nursing, Pharmacology and Physiotherapy, Faculty of Medicine and Nursing, Maimonides Institute of Biomedical Research of Córdoba (IMIBIC) IMIBIC Building, Reina Sofia University Hospital, Av. Menéndez Pidal, s/n, 14004 Córdoba, Spain

**Keywords:** postmenopausal osteoporosis, nursing, prevention, pathophysiology

## Abstract

Osteoporosis is a major public health problem today. We are facing an aging society where the average life expectancy continues to increase. Osteoporosis affects more than 30% of postmenopausal women due to hormonal changes that occur during this time. Postmenopausal osteoporosis is therefore of particular concern. The aim of this review is to identify the etiology, pathophysiology, diagnosis and treatment of this disease and lay the foundation for the role nurses should play in preventing postmenopausal osteoporosis. Several risk factors are associated with osteoporosis. In addition to age and sex, genetics, ethnicity, diet, or the presence of other disorders determine the development of this disease. The key factors include exercise, a balanced diet, and high levels of vitamin D. This is primarily from a solar source, and infancy is the time when future bone formation is greatest. There are now medications that can complement these preventive measures. The work of nursing staff is not only prevention, but also early detection and early treatment. In addition, imparting information and knowledge about the disease to the population is key to preventing an osteoporosis epidemic. In this study, a detailed description is provided of the biological and physiological disease, the preventive measures currently being researched, the information currently available to the population, and how health professionals address osteoporosis from a preventive perspective.

## 1. Introduction

Osteoporosis is defined by the World Health Organization (WHO) as a “progressive systemic skeletal disease characterized by low bone mass and microarchitectural deterioration of bone tissue, with the consequent increase in bone fragility and susceptibility to fractures” [1]. Skeletal fragility can result from not generating a skeleton of optimal mass and strength during growth, either due to a decrease in bone mass with an alteration of the skeletal microarchitecture resulting from excessive bone resorption or an inadequate response to increased resorption during bone remodeling [2]. Osteoporosis is a major public health concern because it is primarily associated with fragility fractures of the hip, spine, and distal forearm. It may increase the risk of falls and increase the risk of fractures [3].

During the twentieth century, there was a large increase in the average life expectancy of different populations worldwide, especially in developed countries. Thus, the average life expectancy in Spain has increased from 30–35 years at the beginning of the 20th century to 83–85 years in the 21st century. In fact, in recent decades, healthy ageing has been prioritized over emphasizing increasing average longevity, with greater interest being given to providing guidelines for the population to achieve a life expectancy closer to the maximum by improving their well-being. As life expectancy increases, the concept of fragility has emerged [4]. Fragility has been defined as “a medical syndrome with multiple causes and contributors that is characterized by decreased strength, endurance and decreased physiological function that increases the vulnerability of an individual to develop greater dependence and/or death”, of which sarcopenia can be an aspect [5]. The prevalence of frailty in the elderly is close to 15%. If this is not addressed, it will become a disability and, ultimately, death, and if treated, the onset of frailty can be delayed. Analysis of the European Union (EU) projected that by 2020, almost 50 percent of those over 70 will be at high risk of impairment [4]. From 15.9 million in 2010 to 21.5 million in 2025, or a 35% rise, the population over 50 is anticipated to grow. Therefore, it is estimated that the total number of fractures will increase from 204,000 in 2010 to 286,000 in 2025, corresponding to an increase of 40% [6].

Economically, it is estimated that 6.6% of men and 22.1% of women over the age of 50 suffered from osteoporosis in the EU in 2010 and 3.5 million fragility fractures were found. The annual direct cost of treating fractures in the EU is around EUR 24 billion, but when indirect costs such as long-term care and treatment to prevent fractures are taken into account, this total rises to EUR 37 billion annually. A British study found that 1 in 2 women over the age of 50 and 1 in 5 men are likely to have an osteoporosis-related fracture in their lifetime. A more comprehensive systematic review published in 2012 used 50 years of literature review and data from the United Nations (UN) organization on demographics. The countries with the highest age-standardized yearly incidence of hip fracture (per 100,000 people/year) were Denmark (57), Norway (563), Sweden (539), and Austria (501). Nigeria (2), South Africa (20), Tunisia (58) and Ecuador had the lowest incidence rates (73). The Russian Federation, Central Europe, Northwest Europe, and Middle Eastern nations such as Iran, Kuwait, and Oman were all considered to be high-risk regions. Taiwan, Singapore, and Hong Kong are other high-risk nations. Low-risk areas in general included Saudi Arabia, Africa, and Latin America (with the exception of Argentina) [7].

Over 204,000 new fragility fractures, including 40,000 hip fractures, 30,000 vertebral fractures, 30,000 forearm fractures, and 104,000 other fractures, are thought to have occurred in Spain in 2010. (i.e., pelvis, ribs, humerus, tibia, fibula, clavicle, shoulder blade, sternum and other femur fractures). Fractures mainly occur due to the coexistence of bone fragility with trauma, usually a fall. Furthermore, the link between bone musculature and bone mass is definitive since one study showed that 75% of people with hip fractures also suffered from sarcopenia. In fact, during the 1-year follow-up period, 56% had at least one fall, 28% had repeated falls, and 12% had a new fracture; 5% of these were hip fractures. (3). It is estimated that approximately 30% of those over 60 years old fall at least once a year, increasing exponentially as they age [8]. In elderly individuals who suffer a hip fracture, it is estimated that more than 20% will die within 6 to 12 months after the fracture, almost 50% will require long-term nursing care, and up to 80% of those who survive will not recover their level of functioning prior to the fracture [9].

The economic burden of fractures and vulnerabilities from previous accidents was estimated at EUR 2842 million in the same year. Accidental fractures accounted for 48% of this cost, long-term care for fractures 37%, and pharmacological prevention 15%. Previous and incident fractures also accounted for 70,800 quality-adjusted life years lost during 2010. According to demographic predictions for 2025, there will be an increase of 82,000 accidental fractures, bringing the total number of fractures to 286,000. Hip, spine (spine), forearm, and other fractures are all thought to have increased by, respectively, 16,700, 11,500, 10,000, and 3500. In 2025, the cost of fractures in Spain is projected to rise by 30% to EUR 3.68 billion [6]. Thus, the substantial societal and personal costs of osteoporosis present problems for public health because the majority of people with the disease do not receive treatment [10]. All these data show the importance of preventing osteoporosis today.

## 2. Pathophysiology

Bone is a living and dynamic tissue in constant formation and resorption to avoid accumulation of micro fatigue fractures, adapt to the mechanical needs of the skeleton, and maintain calcium homeostasis. This phenomenon is known as “bone remodeling” [11]. Bone remodeling is the process of replacing old bone with new bone. The normal process of bone remodeling consists of five stages and requires specialized cells such as osteoblasts and osteoclasts [12]. Usually, the activity of osteoclasts and osteoblasts is in balance and determined by physical and hormonal factors. Thus, the resorption of old bone is carried out by osteoclasts and the formation of new bone by osteoblasts. In a situation of osteoporosis, a loss of balance of the osteoclastic–osteoblastic function occurs, increasing resorption versus bone formation and leading to skeletal fragility due to bone loss [13]. During the activation phase of remodeling, osteoclasts are recruited to the bone surface, subsequently creating an acidic microenvironment between the cells and the bone surface to dissolve or resorb bone mineral content. This process is referred to as the readsorption phase.

Later in the reversal phase, osteoclasts undergo apoptosis and osteoblasts are recruited to the bone surface. In addition, finally, osteoblasts deposit collagen, which will mineralize into new bone in the construction phase. Osteoclasts are derived from hematopoietic stem cells and are closely related to monocytes and macrophages. Tumor necrosis factor (TNF) family member NF-kβ ligand receptor activator (RANKL), as well as the permissive function of macrophage colony-stimulating factor (M-CSF), are required for the differentiation of osteoclast precursors into fully activated multinucleated osteoclasts. Many myeloid cells, T and B lymphocytes, and osteogenic osteoblasts express RANKL, which in turn activates its receptor RANK, which is present in osteoclasts. Following the activation of RANKL, a number of transcription factors and important regulatory enzymes are strengthened to support osteoclast differentiation, proliferation, multinucleation, activation, and survival. Deeply induced bone resorption is the outcome [14,15].

The absence of estrogen during menopause is an important hormonal factor that is involved in this process, since it leads to increased osteoclast activity without a corresponding increase in osteoblastic activity, leading to net bone loss. This process is defined as “decoupling” [12].

On the other hand, osteoprotegerin (OPG), a protein involved in the regulation of bone density, is a natural antagonist of RANKL, which prevents the formation of osteoclasts. However, in women with early menopause, with high levels of RANKL there is an increase in bone resorption and rapid bone loss [14].

## 3. Risk Factors

**Figure 1:**
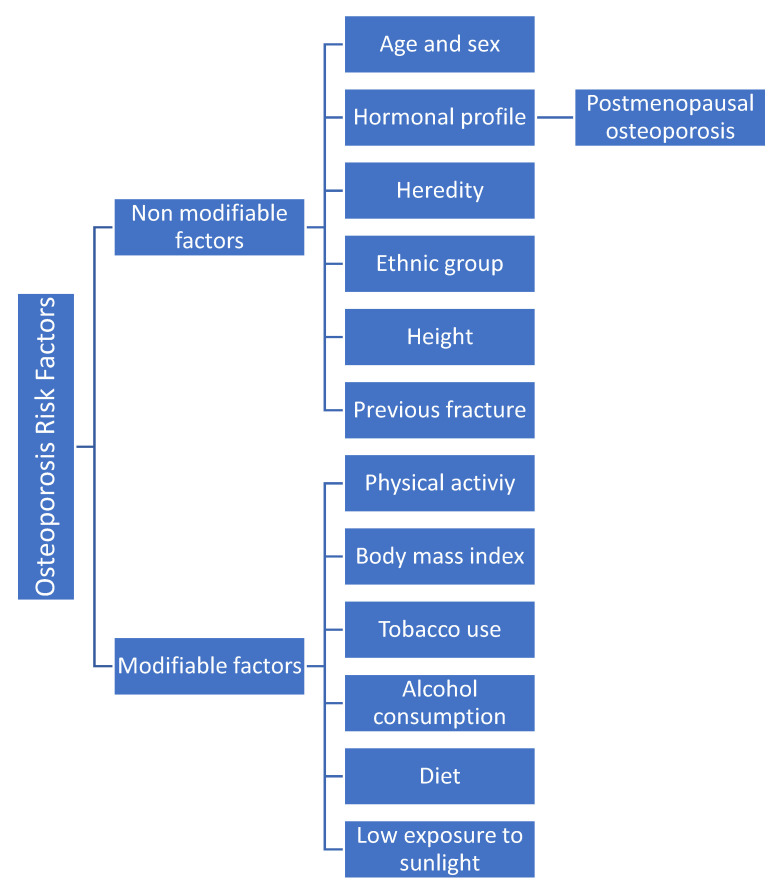
Risk factors for osteoporosis.

Osteoporosis involves many factors that can be divided into non-modifiable and modifiable factors (Figure 1). Alone or synergistically, they may contribute significantly to bone loss leading to osteoporosis. According to recent scientific evidence, poor lifestyle-related factors are also one of the most important factors contributing to the rapid loss of bone density in postmenopausal women [16]. In turn, risk factors can be classified into major and minor risk factors. Major risk factors are those that multiply the risk by 2 or more: age, history of fracture, treatment with corticosteroids, family history of hip fracture, body mass index (BMI) < 20 kg/m^2^, early menopause, and more than two falls in the last year. Minor risk factors multiply the risk by less than 2: active smoking, excessive alcohol consumption (>3 U/day in men and >2 U/day in women), osteopenizing diseases, and treatment with certain drugs (glucocorticoids). The most important risk factor for osteoporosis and fragility fracture is age [11].

### 3.1. Non-Modifiable Factors

#### 3.1.1. Age and Sex

Advanced age increases the risk of low bone density and fractures. Bone density decreases in both sexes as age increases, but bone loss occurs faster after menopause and continues throughout life in women. Women between 60 and 80 years old lose almost twice the bone density as men of the same age [17]. Parallel to bone loss, the risk of fracture is also almost double. Fracture risk is approximately 30% higher in women 80 years or older than in women 50–59 years old. In both women and men, the risk of hip fracture doubles every five years. Men in a particular age group have the same incidence of hip fracture as women in a 5-year younger group [17]. While most women under 50 years old have a normal bone mineral density (BMD), at the age of 80, 27% are osteopenic, and 70% are osteoporotic in the hip, lumbar spine, or forearm. Sarcopenia is also common, with prevalence estimates ranging, according to the definitions, between 9% and 18% of people over 65 years old, increasing up to 30% in those over 80 years old and even more in hospitalized patients [3].

#### 3.1.2. Hormonal Profile

The decrease and subsequent cessation of ovarian estrogen production during menopause lead to increased bone loss, an increase in the first years after menopause is reached. Early menopause is defined as that which occurs before the age of 45, which implies greater bone loss than women of comparable age (from 50–51 years) with normal menopause. This decrease in bone density in women with early menopause leads to an increased risk of osteoporosis-related fractures, being 3 times greater than in women of comparable age with normal menopause. After the age of 70, the importance of this risk factor decreases [15,17].

#### 3.1.3. Postmenopausal Osteoporosis

The maximum bone mass is reached at around 20 years old for the spine and hip, but other bones, such as the radius, reach their peak at 40 years old. During menopause, bone loss accelerates rapidly, starting in the year before menopause and continuing for another 3 years. Between 4 and 8 years after menopause, bone loss is still high [12].

As ovarian function declines during menopause, estrogen production decreases and follicle-stimulating hormone (FSH) levels increase at the same time. The combined effects of estrogen deficiency and increased FSH production significantly stimulate bone resorption, resulting in a period of rapid bone loss that is essential for the development of postmenopausal osteoporosis. Several risk factors are involved in accelerating postmenopausal bone loss [2]. Osteoporosis affects more than 30% of postmenopausal women [18] (Table 1). In 2012, 200 million women worldwide suffered from this disease, of which 20–25% will suffer injuries in the form of a fracture [19]. At present, the risk of fracture for life in women 50 years old is approximately 50% [20]. The osteoporosis screening test is recommended for all women 65 years or older, but only less than a third of women underwent the bone mineral density screening test [18].

Since the 1960s, when the link between menopause and osteoporosis was first established, estrogen treatment has been the standard for preventing bone loss. However, although believed to be valid, there were no fracture data [12]. Today, we find a much more complete treatment, as described in this review.

#### 3.1.4. Heredity

Women who have a mother with osteoporosis are slightly more likely to develop it. In fact, a daughter’s risk is moderately increased if the mother has a hip fracture. There is some evidence that maternal hip fractures increase the risk of spinal compression in offspring. On the other hand, twin studies have yielded inconsistent results [17].

#### 3.1.5. Ethnic Group

Hip fractures are most common in white women and least common in African American women. A recent British study found that black individuals had the lowest fracture rates. The fragility fracture rate for white women was 4.7 times higher than for black women and 2.7 times higher for white men than for black men. Mixed ethnicity or South Asian ancestry had hip fracture rates less than half that of Caucasians [7].

The incidence of hip fractures varies greatly by region and race, and can vary significantly within countries. In Europe, epidemiological studies have reported up to seven-fold differences in incidence between countries. In white populations, the hip fracture rate, adjusted by age and sex, is usually higher than in black or Asian populations. Depending on the fracture rate adjusted to age, the highest are concentrated in Sweden and the north of the United States, intermediary rates are found in the Asian population and the lowest rates in the African population [3]. Some racial differences can be explained by differences in adjustable lifestyle aspects such as low milk intake, smoking, lack of sun exposure, low BMI, and low physical activity. Genetic factors also play a role. However, the relationship between history of fragility fractures and the falls is more unpredictable. For example, vertebral fractures can occur spontaneously and are associated with heavy lifting or bending rather than falls. When comparable methods and definitions were used in studies, the prevalence of vertebral fractures was more similar than that of hip fractures across all regions. 

Although there is a geographical variation in the rate of distal fracture of the forearm, less than 20% of patients with forearm fractures are hospitalized, and this number varies dramatically around the world, partly explained by case determination methodology. It may be a logical consideration. As noted above, in addition to fracture outcomes, numerous studies have shown that loss of muscle mass leads to fragility and loss of independence. Overall, it has been proposed that normal operation is affected when there is a 30% loss, leading to a system failure when the loss reaches 70%. Thus, the greater muscle mass found in some breeds protects against sufficient muscle loss leading to loss of vulnerability and independence, given similar rates of muscle loss in all groups. Alterations in body composition are well reported, which explains the greater muscle mass in black populations [3,21].

#### 3.1.6. Height

Tall women have a greater risk of osteoporosis and fracture. However, scientific evidence is poor that demonstrates that the height of the body at the age of 25 can predict a subsequent fracture [17]. In epidemiological studies, an association between body weight at one year of age and the development of osteoporotic fractures in adults has been demonstrated. In fact, in a longitudinal study developed Den Helsinki (Finland), a correlation between child growth and the risk of hip fracture in adults was established [3].

#### 3.1.7. Previous Fracture

A major risk factor for the development of a fracture is the history of a previous fracture at a specific osteoporosis point. Fracture risk approximately doubles in the presence of a prior fracture, including morphometric vertebral fractures, increasing when there is more than one vertebral fracture. The risks are partly independent of the BMD [1]. Thus, in the case of hip fractures, it more than doubles the risk for those who have already suffered a fracture of this type [17]. Another risk factor, in people over 75 years of age, is poor vision, which is considered an independent risk factor for falls and fractures. In fact, in women with a mean age of 80 and poor vision, the risk of hip fractures is five times higher. Therefore, poor vision is a major risk factor for accidental falls leading to hip fractures in both sexes [17]. 

### 3.2. Modifiable Factors 

#### 3.2.1. Physical Activity

Physical inactivity is an independent risk factor for hip fractures in both women and men. So, the percentage of physically inactive individuals increases with age, with older women being more inactive than older men. The absence of engaging in weight-bearing muscle-strengthening exercises increases the risk of hip fracture [17,22]. 

As compressive force increases, bone density rises in response to physical and mechanical stress, and bone mass and density increase in response to increased loading. Thus, there are two forces that act on the bone, muscle contraction and gravity. Absence of adequate mechanical stimulation leads to bone loss that is mainly mediated by a proportional increase in bone resorption without an increase in bone formation. Contrarily, exercise stops the loss of bone density. However, a recent meta-analysis found no effect on bone mineral density in the forearm or femoral neck, but identified a significant protective effect of exercise on the lumbar spine. Indeed, weightlifters and other sportsmen benefit from mechanical stress on their bone mass. This increase could be concentrated on the side of the burden, such as a tennis player. 

On the other hand, immobilization can be associated with rapid bone loss, and prolonged immobilization may lead to fractures, as in patients with paraplegia or hemiplegia. Inactivity has been shown to reduce muscle mass and strength at all ages. Bedridden studies have shown that muscle strength declines before muscle mass declines. Likewise, physical activity has been reported to have a significant impact on muscle mass and strength. In fact, increasing recreational physical activity in middle age has been shown to reduce the risk of movement disorders in older adults [3,23]. 

Finally, it is worth mentioning a study conducted in the United Kingdom considering that socioeconomic prosperity is an important factor leading to lower levels of physical activity and a higher probability of falling onto hard surfaces (in fact, a gross domestic product (GDP) GBP 10,000 higher per capita was associated with a 1.3% increase in the probability of hip fracture) [7].

#### 3.2.2. Body Mass Index (BMI)

Low BMI is a well-established risk factor for decreased bone density and future fractures [24].This risk is more pronounced in lean individuals with a BMI below 20 kg/m^2^. The association between fracture risk and thinness is highly dependent on BMD [25]. The risk of hip fracture after BMD adjustment is low. In this sense, a protective effect of obesity on bone mass has been described through various mechanisms. These include skeletal-loading mechanical and hormonal factors, primarily associated with increased peripheral estrogen production. In later menopause, adipose tissue is responsible for most of the circulating estrogen due to the conversion of androgens to estrogens. Over time, body fat increases, muscle mass decreases, changing body composition, and consequently we can define it as sarcopenic obesity. These individuals often have more muscle mass than non-obese adults, but less lean body mass compared to total body weight. Obesity is associated with inflammation and may play an important role in the process that leads to sarcopenia. Indeed, longitudinal studies have described that remarkable weight loss is associated with a faster decline in grip force, possibly reflecting confounding comorbidity [3].

#### 3.2.3. Tobacco Use

The inverse association between smoking and bone density is well established and is due to multiple factors, including early menopause, weight loss, and increased metabolic breakdown of exogenous estrogens [3]. Smoking cessation should be advocated in patients with postmenopausal osteoporosis or at risk of suffering it. Cigarette smoking reduces BMD, increases estrogen metabolism, leads to early menopause and malnutrition, and causes lung damage [13].

#### 3.2.4. Alcohol Consumption

It is well known that excessive alcohol consumption negatively affects skeletal health, highlighting its adverse effect on calcium metabolism, along with its toxicity on osteoblasts, protein metabolism, and gonadal function. On the other hand, the risk of fracture increases with high alcohol consumption [3,26].

#### 3.2.5. Diet

Diet has a significant effect on the health of bones and muscles in later stages of life. The intake of calcium, vitamin D, and protein in the diet not only of the elderly, but also in children since childhood can play a role in the development and maintenance of maximum BMD [27]. Meanwhile, other substances such as caffeine or caffeine salt can have adverse effects on the bones, possibly by increasing urinary excretion of calcium directly and therefore contributing to a negative calcium balance. Likewise, nutrition plays an important role in the pathogenesis of muscle loss [3].

#### 3.2.6. Low Exposure to Sunlight 

A Swedish study found that hip fractures increased with northern latitude and showed seasonal variations. One reason for this is that the sun is lower in Nordic countries, which can lead to lower levels of vitamin D and an increased risk of osteoporosis and hip fractures [17].

## 4. Comorbidities and Pharmacotherapy

Several disorders are associated with osteoporosis, sarcopenia, and increased susceptibility to fragility fractures. These include rheumatic diseases such as rheumatoid arthritis and ankylosing spondylitis, immobility, low BMI, risk factors as hyperparathyroidism, endocrine disorders (with bone loss), and malabsorption syndromes such as celiac disease. Lean body mass loss and a number of chronic disorders, including cancer, heart failure, and chronic obstructive pulmonary disease (COPD), are linked.

Acute hypercatabolism and elevated production of pro-inflammatory cytokines cause these illnesses. Cachexia is a condition that can affect persons of any age, but is more prevalent among the elderly. Yet, a variety of age-related disorders and normal aging also cause a rise in the production of these pro-inflammatory cytokines, namely IL-6, IL-1, and TNF [3].

Glucocorticoids are an important cause of osteoporosis and fractures. Bone loss is thought to be more rapid in the first months of treatment, although the bone loss is more marked in the spine, where spongy bone predominates. The risk of fracture conferred by the use of corticosteroids is not mediated solely through their effect on BMD. In a meta-analysis, the range of relative risk for hip fracture was 4.4-fold to 2.5-fold, with higher risk at younger ages. This increased risk changed immediately after BMD adjustment and was independent of previous fractures. Similarly, in clinical practice, muscle weakness is commonly observed in patients receiving oral glucocorticoids, although muscle weakness in this group has not been well studied [3].

A daily dose-based treatment (tablet) equivalent to at least 5 mg of prednisolone reduces bone density and increases fracture risk. Prednisolone 7.5 mg once daily for 3 months doubles the risk of hip fractures and nearly triples the risk of spine fractures [17].

## 5. Diagnosis of Osteoporosis

When fragility fractures or bone mineral density, as determined by a bone densitometer, are 2.5 standard deviations (SD) below the young adult reference group, osteoporosis is clinically diagnosed [12]. The predictive value of BMD for hip fracture is at least as good as that of blood pressure for stroke [1]. By convention, the value is described as a T-score [12].

The four general diagnostic categories for women are as follows, according to the WHO criteria,: normal, with a BMD of less than 1 SD below the reference population (T-score > −1); osteopenia, with a BMD higher than 1 SD below the reference population but less than 2.5 SD (T-score −1 and >−2.5); osteoporosis, with a BMD of 2.5 SD or higher (T-score > −2.5) below the reference population, severe osteoporosis [28].

Tests may be performed once every 2 years, although more frequent tests are allowed if considered medically necessary. The evaluation of the BMD by using dual-energy X-ray absorptiometry (DXA) in the hip and spine, also known as central DXA, is the gold standard method for diagnosing osteoporosis. DXA central scans involve low radiation exposure and are easy to perform, even if the patient is in bed or has mobility problems [13].

Although low BMD identifies individuals at high risk of fracture, most fragility fractures occur in individuals with less severe loss of bone mass or normal BMD. This consideration makes it more practical to delineate microarchitectural parameters using high-resolution peripheral quantitative computed tomography and to combine BMD with clinical risk factors that are partially independent of BMD in an absolute risk calculator. A more detailed assessment of the bone itself was performed, including a systematic approach, such as the FRAX algorithm [7].

The WHO-sponsored FRAX fracture risk assessment tool integrates BMD in the femoral neck (or total hip) with a number of clinical risk indicators for fracture that are mainly independent of BMD. It is helpful in determining the likelihood of hip fracture and significant osteoporotic fractures, such as clinical fractures of the spine, hip, proximal humerus, and distal forearm, in people between the ages of 40 and 90 who have not previously undergone therapy [29].

The lack of detection of clinical osteoporosis when present probably contributes to the current lack of awareness of the consequences of this disease among both doctors and patients, has an impact on the repayment strategies of payers, and influences those responsible for the formulation of policies in the public health sector by underestimating the number of people at high risk of fracture and affecting the design of clinical trials of new agents to reduce the risk of fracture. It has been recommended to formally expand the criteria to allow the diagnosis of osteoporosis to include the presence of certain low trauma fractures or the determination of a high risk of fracture using FRAX, without a T-score of −2.5 or less [29].

The guidelines recommend screening women aged 65 and older and men aged 70 and older. However, all postmenopausal high-risk women should be examined. A recent model suggests that starting the evaluation at age 55 years in postmenopausal women may be more cost-effective than current guidelines. The most important factors in determining the optimal screening intervals appear to be the score and age. For older postmenopausal women with normal BMD or mild osteopenia at the start of the study, we can wait up to 15 years before repeating the test. Older postmenopausal women with moderate osteopenia at baseline can be examined every 5 years, and those with advanced osteopenia should probably be examined annually [30].

## 6. Prevention, Treatment, and Nursing Care in Patients with Osteoporosis

The best strategy for the treatment of osteoporosis is prevention. In order to acquire an acceptable BMD and constantly preserve bone health, it is essential to encourage bone development in children and then to lessen the loss of bone mass as people age [11]. To achieve these objectives, a series of guidelines are needed that help bone health to the maximum.

### 6.1. Physical Exercise

Exercise can prevent osteoporosis 5 times more among physically active women than women who are physically inactive and immobile [18]. It is necessary to exercise and perform physical activity regularly for a healthy bone, avoiding a sedentary lifestyle. If the bones are not energized and physically active, the osteocytes do not receive signals about the need for remodeling, removal of damaged bone, and synthesis of new bone. Therefore, a gradual reduction in total bone mineral density occurs. In postmenopausal women, physical activity or physical exercise should provide the voltage necessary to maintain bone density [16].

Physiologically, almost immediately after starting physical exercise, the oxygen absorption of the whole body is multiplied by 20 during maximum exercise, the heart rate increases up to 200 beats per minute, and ventilation increases up to a maximum of about 150 L per minute. The whole body’s metabolism responds to exercise by increasing the absorption of muscle glucose, glycogenolysis, and the renewal of adenosine triphosphate (ATP). Skeletal muscle is a key player in exercise generating dramatic changes in metabolism, including active glycolysis, turning muscle into tissue that produces large amounts of lactic acid, increased use of glucose and oxygen, and production of carbon dioxide [4]. Physical activity has proven to be an effective non-pharmacological approach to improve bone mass, but today, we find various exercises valid for this purpose [31].

The mixed-impact loading programs reported to be most effective in preserving BMD in the lumbar spine and femoral neck were those that combined low- to moderate-impact exercises, such as jogging, walking, and stair climbing, with resistance training. However, high impact and more demanding exercises were ineffective [32].

A randomized controlled trial in women with established osteoporosis showed that a 20-week community exercise program conducted by a certified fitness trainer improved participants’ dynamic balance as measured by speed on an 8-way circuit. It also improved knee extension strength, an independent risk factor for fall and osteoporotic fractures. There was also a clear trend towards lower balance (a measure of static balance) for women in the intervention group, but it was not significant. Therefore, the intervention showed no adverse effects and improved dynamic balance and knee extension strength, at least two important risk factors for falls [33]. A physical training program based on low-intensity balance and strength training, performed in 1-h sessions 3 days a week for 6 months, using simple, readily available and inexpensive materials to improve upper and lower extremity muscle strength, produced significant improvements in the static and dynamic balance of women with postmenopausal osteoporosis, compared to those who did not participate in any exercise program [34]. The National Osteoporosis Foundation lists walking as one of the most effective exercises for postmenopausal women to maintain or increase bone mineral density. It was shown that older postmenopausal women who participate in recreational sports or walk for 30 to 60 min more than twice a week have a lower risk of osteoporosis and fractures. Self-weight or resistance training promotes bone density effectively, and aerobic exercise improves muscular balance and activity, lowering the risk of falls [16]. The above indicates that the multi-component workout is the most efficient. Strength training, coordination, and balancing exercises are all part of this strategy. Yet as of right now, there is no multi-component fitness regimen that can be stratified to defend against frailty-related issues as well as social and cognitive components to enhance elderly people’s quality of life. Globally, significant efforts are being undertaken to create an effective fitness regimen [4].

It should be noted that exercise performed in an aquatic environment, often known as aquatic exercise, presents lower risks of traumatic fracture and the joints are exposed to less stress and impact (through the reduction of the load due to buoyancy) compared to exercise on land, such as running, resistance training and strength training. Furthermore, aquatic exercise has been highly recommended for the elderly, especially for those with disabilities, due to the reduction in pain and the increased safety; it also provides additional benefits for neuromuscular and functional health and cardiometabolic health. Given the lower prevalence of pain and injury, dropout rates for water exercise are likely to be lower than for some land-based activities. Moreover, some older adults may simply enjoy aquatic exercise or wish to participate for social reasons [31].

Hence, rather than an increase in bone size, any gain in bone strength is mostly caused by an increase in tissue density, a decrease in endocortical bone loss, or both. These findings suggest that given the significance of cortical thickness and bone size for fracture risk, the years of growing may be the most suitable period to increase bone strength and lower the risk of fracture in the elderly [32].

### 6.2. Diet

Adequate nutrition providing the essential nutrients for the bone is needed to prevent osteoporosis. However, it must be considered that both favorable and unfavorable nutrients exist for bone health.

#### 6.2.1. Nutrients Favorable for Bone Health

##### Calcium and Vitamin D

Calcium provided by the diet is essential to achieving correct mineralization of the bone and maintaining its quantity and quality. However, calcium has multiple cell biological functions that are crucial for the proper functioning of the organism, so it must always be maintained within a minimum concentration range in the extracellular environment. To ensure that dietary calcium insufficiency does not impair cellular biological functions, calcium mobilization mechanisms are initiated from bone deposits to maintain normal extracellular levels, at the expense of bone mass quantity or density and structure or quality of the bone. Therefore, it is recommended to increase calcium intake through food or, if necessary, with supplements to reach 1000–1200 mg daily [11,35]. There is controversy about the possible increase in cardiovascular risk associated with calcium supplements, particularly if the recommended daily dose is exceeded or high-dose supplements are administered. Until this aspect is clarified, it may be prudent to try ensuring that the contribution comes mostly from the diet; and in the case of using calcium supplements, avoid doses higher than 500 mg in each administration. Virtually all clinical trials conducted with osteoporosis drugs have routinely included the joint administration of calcium and vitamin D supplements [11]. Combined calcium and vitamin D supplementation is significantly associated with a decrease in total and hip fractures in several populations [36]. Therefore, the Osteoporosis Guidelines recommend their combined use; and the technical data sheets of these drugs include that if the calcium intake in the diet is inadequate, patients should receive a complementary contribution of calcium and vitamin D. Dairy products (milk, yogurt, cheeses, etc.) are excellent dietary sources of accessible calcium. Yogurt has a high nutritional value, and provides a good amount of calcium, vitamins of groups A and B, and different minerals. In addition, compared to milk, yogurt has a higher amount of calcium and proteins of high biological value [11]. The consumption of yogurt has multiple benefits associated. For instance, its partially denatured proteins are more digestive than milk proteins; therefore, yogurt can be very interesting in feeding children and the elderly. A single serving of yogurt provides 270 to 450 mg of calcium (depending on the yogurt type), comprising almost half of the daily needs. Yogurt helps maintain the health of bones and teeth and achieve an optimal balance in the intestinal flora, which favors digestive processes and recovery after an episode of diarrhea, even that which frequently occurs by taking antibiotics. Yogurt favors the correct absorption of nutrients and vitamins, especially vitamin K, vitamin B12, and folic acid. A study conducted on women over 58 years old who ingested daily a fermented milk product enriched with high doses of vitamin D showed a significant improvement in vitamin D levels [11].

Vitamin D is essential for calcium absorption and metabolism; its deficiency is a decisive factor in the development of osteoporosis [37]. When the intestinal absorption of calcium is decreased, the secretion of parathyroid hormone (PTH) is stimulated, causing secondary hyperparathyroidism to obtain calcium from the bone and take it to the extracellular environment. This leads to a more fragile bone that is more susceptible to fractures, even with “low impact” trauma. Vitamin D is involved in calcium homeostasis as well as influencing muscle tone and muscle contraction. Vitamin D deficiency leads to muscle weakness, an increased tendency to fall, and an increased risk of fractures. Moreover, it seems that vitamin D insufficiency favors the development of some types of cancer such as breast, prostate, and colon cancer, the incidence of inflammatory, autoimmune or metabolic diseases, and susceptibility to infections, hypertension, or dementia, in addition to increased global mortality. The body obtains 90% of vitamin D from exposure to the sun and 10% from the diet [11]. A prospective study on the intake of calcium, vitamin D, milk intake and hip fracture among postmenopausal women showed that exposure to sunlight can prevent the risk of developing osteoporosis by 12.5 times [18]. A sufficient amount of ultraviolet (UV) type B photons must reach the epidermis, so that the cutaneous synthesis of vitamin D becomes effective. This explains why in latitudes north of the 35° parallel (almost all of Spain), vitamin D is not synthesized during the winter months. In addition, other factors limit skin synthesis, such as skin aging or sunbathing with protective creams. Even with a healthy diet, it is difficult to obtain a vitamin D intake of more than 200 international units (IU) (5 μg) every day, far from the 800 IU (20 μg) per day that tends to be recommended today. Therefore, there is a high prevalence of vitamin D deficiency (blood levels < 30 ng/mL), which affects more than 50% of the Spanish population and exceeds 70% of women after menopause [11]. 

In Spain, a very limited number of foods contain vitamin D, such as fatty fish (such as salmon and mackerel) or mushrooms, whereas dairy products and eggs contain only small amounts of vitamin D [5] (Table 2). The consumption of 1200 mg of calcium per day, together with 800 IU of vitamin D has shown to be effective in treating and preventing osteoporosis. When in doubt about administering vitamin D, clinicians recommend that it should be administered only in case of deficiency (<20 ng/mL) or deficit (<30 ng/mL) [11] (Table 3).

##### Vitamin K

Vitamin K is an essential factor in the coagulation process and in the activation of bone proteins such as osteocalcin (the most abundant in bone), osteoprotegerin, and RANKL, which are of great importance in the activity of osteoclasts and bone health. Vitamin K deficiency produces anomalous osteocalcin (infracarboxylated), and is a predictor of bone fracture risk. In bone metabolism, vitamin K has a synergistic action with vitamin D. Vitamin K is found in green vegetables, such as broccoli, brussels sprouts, or spinach; also in vegetable oils and cereals; and in smaller amounts in dairy, meat, and fish [11]. Natto, a fermented soy product, is the richest dietary source of vitamin K, and vitamin K is also found in aged and curdled cheeses [38].

#### 6.2.2. Proteins

The recommended dietary protein intake for the general moderately active population is 0.8 g protein per kg body weight per day. However, due to the increased protein requirements of healthy older adults, the European Society of Clinical Nutrition and Metabolism suggests a recommended daily allowance of 1.0 to 1.2 g/kg body weight per day, which is optimal for healthy older adults. Data from the National Health and Nutrition Examination Survey (NHANES) show that despite recommendations, daily protein intake is declining among older adults, with at least 8% of women consuming inadequate amounts of protein. Overall, 35% of institutionalized older adults do not reach the recommended daily dose. Protein intake increases plasma levels of IGF-1, a hormone recognized as essential for muscle growth and regeneration, along with muscle mass and strength. Additionally, protein functions by regulating the absorption of calcitriol and intestinal calcium, thereby improving bone health [5].

#### 6.2.3. Other Compounds in the Diet

**Phytoestrogens** are found in some plant foods, especially soy isoflavones. They have a great affinity for β-estrogenic receptors, mimic the action of estrogens, and are a therapeutic alternative to them. Isoflavones can be found in cherries, oranges, grapes, and soy. They exert their effect on the formation and mineralization of bone by stimulating the action of osteoblasts and inhibiting that of osteoclasts by inducing their apoptosis. Several studies have shown that isoflavones improve bone health [11].

**Omega 3 polyunsaturated fatty acids** (PUFA) are also related to osteoporosis. A diet with a low ratio of omega-6/omega-3 PUFA in youth is associated with bone gain and a high peak of BMD. In contrast, a diet with a high ratio of omega-6/omega-3 PUFA is associated with a lower hip BMD in adults over 45 years old in both sexes [11].

**A high intake of potassium in the diet or potassium supplements** improves calcium balance and reduces bone resorption in the short term (3 to 6 months), but no completed long-term studies are available [11].

The **consumption of water** rich in bicarbonate has a more favorable effect on bone turnover than that of water richer in calcium but poor in bicarbonate. Therefore, taking advantage of the increase in mineral water consumption in industrialized countries, promoting the use of mineral waters rich in bicarbonate and calcium and low in sulfates, constitutes an opportunity for bone health [11].

### 6.3. Nutrients Unfavorable for Bone Healths

**Salt intake** is associated with increased urinary calcium loss. Four weeks with an elevated sodium diet (>15–17 g/day of salt) is enough to cause a significant increase in bone resorption in women after menopause. Reducing sodium intake to <5–6 g/day in the usual diet is very important, especially limiting prepared foods. The addition of potassium citrate could prevent urinary calcium loss and excessive bone resorption [11].

**Caffeine** from coffee can increase the excretion of calcium in the urine, and even 24 h later, this loss cannot be fully recovered. Coffee consumption reduces the absorption of interstitial calcium, and high dosages (>300 mg/g or 4 cups per day) can hasten bone loss at the level of the lumbar spine in older postmenopausal women [16]. Indeed, women who drink coffee or strong tea regularly have noticed fewer benefits of applying a calcium-rich diet [19].

**Vitamin A** is necessary for bone health and growth; however, when consumed in high doses (>1500 μg of retinol or equivalent), vitamin A stimulates osteoclasts and inhibits osteoblasts, increasing bone remodeling and decreasing BMD. An elevated serum level of vitamin A (>2.26–2.40 mmol/L) inhibits the effect of vitamin D and increases the risk of fracture. In Spain, there is a high association between low levels of vitamin D (>70% in postmenopausal women) and high levels of vitamin A. Therefore, the safety of current nutritional supplements with vitamin A should be reconsidered, at least concerning osteoporosis and the risk of osteoporotic fracture [11].

**Moderate protein intake**, as discussed above, guarantees the proper functioning of the entire organism. In contrast, a high protein intake can be harmful: excess protein in the diet predisposes bone decalcification, with consequent disorders in the metabolism of calcium, phosphorus, and vitamin D and causes kidney disease [11].

## 7. Factors Unfavorable for Bone Health 

### 7.1. Tobacco Use

Smoking influences skeletal metabolism through direct toxic effects on bone tissue and indirectly through the hormonal system. Bone density is lower among smokers and former smokers. This applies to both sexes. The risk of hip fracture among women who smoke is up to three times higher than among non-smokers. The largest risk is found among low-weight women. Although quitting smoking will reduce the risk, a high risk will persist [17]. In a recent study, the authors established that although they could not demonstrate a significant difference in bone density at 50 years old between smokers and non-smokers, bone density in women decreased by an additional 2% for every 10 years increase, with a 6% difference at 80 years old. Despite mentioning confounding effects of BMI and estrogen, this meta-analysis could not fully address lifestyle differences between smokers and nonsmokers. Another recent meta-analysis focused on smoking and discovered that current smokers had a considerably higher fracture risk than nonsmokers. Men and women had significantly different risk ratios for fractures of all types and osteoporotic fractures, but not for hip fractures. Just 23% of the risk of hip fractures linked to smoking was explained by low BMD. Nonetheless, the risk of all fracture consequences was somewhat reduced after BMI normalization. Those who currently smoked had a significantly higher risk of fracture than those who had never smoked, whereas those with a history of smoking had a much higher risk than those who had never smoked. Unfortunately, there exists little research that has examined the connection between smoking and muscle mass, despite the fact that it has frequently been linked to a low BMI and little physical activity [3]. 

### 7.2. Alcohol Consumption

Excessive alcohol consumption has a negative impact on the mechanism of bone remodeling, proliferation, and osteoblastic activity and, therefore, a direct negative effect on bone homeostasis [16]. A study developed by Curtis et al., described no significant increase in the risk of fracture with consumption of 2 units or less daily [3]. However, the intake of 3 or more units daily in men and 2 or more in women is associated with an increase in the risk of dose-dependent fracture [16]. Excessive alcohol consumption leads to low muscle mass due to the associated effects of deprived nutrition, low levels of physical activity, and hormonal irregularities [3]. People who consume a lot of alcohol have five times higher risk of hip fracture than those who abstain. Women with high alcohol consumption have a 40% increase in the risk of hip fracture [17].

### 7.3. Pharmacological Treatment

Several pharmacological interventions decrease fracture risk in postmenopausal women with osteoporosis [1]. Antiresorptive pharmaceuticals, which decrease bone resorption, and anabolic drugs, which increase bone growth, are the two categories of osteoporosis treatments. There are currently a number of osteoporosis therapy methods that effectively lower the risk of hip, non-vertebral, and vertebral fractures. To register any new osteoporotic agent, there must be unequivocal proof that it lowers the risk of vertebral fracture. The largest class of antiresorptive medications is made up of bisphosphonates due to their strong affinity for bones and extensive safety track record. In most situations, alendronate or risedronate are the first-line medications. The best choices for women who cannot take or have contraindications to oral bisphosphonates are intravenous bisphosphonates or denosumab [14]. However, recently, the Endocrine Society included guidelines for the use of Romosozumab in postmenopausal osteoporosis. Romosozumab is a monoclonal antisclerostin antibody, which has a dual effect, increasing bone formation and decreasing bone resorption, by blocking the sclerostatin pathway [39]. Different studies about the efficacy of the blunt observe a significant improvement in patients regarding bone mineral density of the lumbar spine, the total hip and the femoral neck during a period spanning between 12 and 36 months. In addition, it is shown to be effective in significantly reducing the risk of fractures and falls in postmenopausal women with osteoporosis [40].

#### 7.3.1. Alendronate

In postmenopausal women with osteoporosis, alendronate 10 mg once daily reduces hip fractures, vertebral fractures, and non-vertebral fractures. The primary adverse effects of alendronate include upper gastrointestinal problems, intestinal issues, migraines, and musculoskeletal pain. This must be taken after a night of fasting and at least 30 min before the first meal, drink, or medication (other than water) of the day (including calcium) [1].

#### 7.3.2. Risedronate

When taken daily at a dose of 5 mg by postmenopausal women with osteoporosis, risedronate reduces both vertebral and nonvertebral fractures. In a large group of elderly women, risedronate dramatically reduced the risk of hip fractures; this effect was stronger in osteoporotic women. Headache and musculoskeletal discomfort are a few of the side effects, along with symptoms of the upper digestive tract and intestinal issues [1].

#### 7.3.3. Ibandronate

Oral ibandronate showed a significant decrease in vertebral fractures at a dose of 2.5 mg daily. In an analysis of high-risk women (BMD T score in femoral neck less than −3.0), non-vertebral fractures were significantly reduced. No data are available for hip fractures. Based on BMD studies, approval for oral formulations of 150 mg once a month and 3 mg intravenously every 3 months was granted. Side effects of oral preparation include upper gastrointestinal side effects and intestinal disorders. Ibandronate (oral) should be taken after an overnight fast and 1 h before the first meal or drink (other than water) of the day or any other oral medication or supplement (including calcium) [1].

#### 7.3.4. Denosumab

Denosumab is a fully humanized monoclonal antibody against the receptor activator of the nuclear factor Kappa B Ligand (RANKL). It is approved for the treatment of hormonal ablation-associated bone loss in men with prostate cancer, who have a higher risk of fractures, as well as the treatment of osteoporosis in postmenopausal women who are at an increased risk of fractures. A 60 mg subcutaneous injection is given once every six months. In postmenopausal women with osteoporosis, denosumab lowers the risk of hip fractures, non-vertebral fractures, and vertebral fractures [1].

Current antiresorptive drugs are effective, but some are limited by side effects, concurrent comorbidities, and inadequate long-term compliance [14]. However, over 10 years of treatment, there are no data to guide judgments; therefore, each patient’s treatment options should be carefully reviewed [1]. 

#### 7.3.5. Romosozumab

The SOST gene encodes the protein sclerostin, which inhibits osteoblastogenesis and prevents the production of new bone by disrupting pathways important for bone morphogenesis and development, such as the Wnt signaling pathway [41,42,43], essential for skeletal development, skeletal homeostasis, and bone remodeling [42]. Therefore, blocking the action of sclerostin would favor increased bone formation and decrease resorption. This would therefore be the mechanism of action of Romosozumab [39]. 

The administration of Romosozumab would not only increase bone formation by acting on Wnt, but would also favor the recruitment of osteoprogenitor cells, increasing bone mass [44]. In fact, authors such as Singh [40] have recently reviewed the scientific evidence on the use of Romosozumab, concluding that there is moderate-quality evidence that favors the use of Romosozumab in postmenopausal osteoporosis. In this sense, the recent update of the Endocrinological Society establishes that “women with severe postmenopausal osteoporosis and at very high risk of fracture (defined as T-score less than −2.5 and a prior fracture) or with a history of multiple vertebral fractures should be given Romosozumab 210 mg monthly for up to one year to reduce the risk of vertebral, hip, and nonvertebral fractures and in women who have completed the course of Romosozumab, should be treated with antiresorptive therapies in order to maintain bone mineral density gains and reduce future risk of fracture” [45].

### 7.4. Hormonal Treatment

As mentioned above, the absence of estrogen during menopause accelerates bone loss that begins 2–3 years before the last menstrual period and continues up to 5–10 years. As we have commented, the loss of estrogen is associated with the loss of the balance of osteoclastic–osteoblastic activity due to an increase in the useful life of osteoclasts and a decrease in osteoblasts. Furthermore, it is also associated with the presence of cytokines such as TNF-α among others. These cytokines expand the set of osteoclast precursor cells and increase the expression of the key molecule that regulates the development, activity, and lifespan of osteoclasts, the activator of RANKL, through osteoblasts and other cells in the bone microenvironment. This suppression of osteoclastic activity by estrogen replacement therapy has been used effectively for decades and was the pillar of the prevention and treatment of postmenopausal osteoporosis. Treatment of mild to severe menopausal symptoms is currently the principal indication. The impact of hormone replacement treatment (HRT) on postmenopausal women was examined in a meta-analysis of 57 randomized placebo-controlled trials. It showed that the decrease in estrogen caused bone loss at a rate similar to that seen in early menopause, and that estrogen was much more effective than a placebo at maintaining and raising BMD. The incidence of both vertebral and non-vertebral fractures was found to be lowered by HRT in the same study [20].

Estrogen-based HRT plays an important role in maintaining and improving muscle mass and strength, and protecting against muscle damage. The skeletal muscle benefits of estrogen and its additional beneficial effects on bone and metabolic health in older women provide additional incentives for the use of HRT to improve overall health in postmenopausal women. Regarding associations with improvements in contractile function and muscle strength in women aged 50 to her 65, studies in older postmenopausal women are inconclusive. A systematic review of long-term HRT in perimenopausal and postmenopausal women found that HRT is effective in preventing osteoporosis but is only recommended as an option when the risk of developing the disease is so high that other strategies are not available. It was concluded that the introduction of HRT, if needed, should be short-term, provided it does not increase the risk of cardiovascular disease, thromboembolic disease, and various types of cancer. However, the disadvantages associated with menopause do not necessarily occur in all women at the same time. Sometimes, there are only some adverse effects and other times these shortcomings end up together and are very obvious and frustrating. Currently, there is much debate about the risks associated with taking HRT, and in this sense, the latest scientific evidence suggests that menopause can be managed by adopting healthy habits that prevent associated disorders [5].

### 7.5. Health Information and Education 

Osteoporosis is a reality that women are familiar with but at a distance. Information about osteoporosis obtained in the media or from family and friends does not necessarily make them worried. Women feel young, with a healthy lifestyle, and do not perceive that their bones require care. Thus, osteoporosis is not perceived as a disease but rather a natural deterioration of the bones, an inevitable part of aging [46]. Moreover, even though the prevalence of the condition is widely known, nothing is known about its causes, treatments, or how serious its effects will be. A common knowledge of the value of calcium intake and exercise is also present. Other risk factors, such as smoking, vitamin D insufficiency, or ethnicity, are, however, little known. Women are unlikely to take action to lower their risk of getting osteoporosis, according to the apparent lack of information about the severity of osteoporosis and insufficient knowledge of preventative actions [47].

In a study conducted on women between 20 and 49 years old, most of them with university studies found that younger women (20–29 years old) show the lowest level of knowledge. Furthermore, in general, they consider the disease as “serious” but not as “disabling”, in addition to the low susceptibility. They are aware of the benefits of calcium intake, although they agree that calcium-rich foods contain excessive amounts of cholesterol. Another important fact is that older women are more motivated to take care of their health than younger women [47]. Elements that can prevent the effective attention of postmenopausal osteoporosis are identified. Despite many information campaigns, studies on the complications of the disease, and national and international guidelines on treatment indications, there are still strong barriers to starting or continuing treatment [46]. The goal for osteoporosis prevention programs is to urge women from adolescence to premenopause to adopt risk reduction practices and to identify risk factors early [47]. Once known the beliefs women have today about the disease, nursing professionals know on which points they should act, showing the susceptibility and seriousness of osteoporosis, food options with high calcium content that do not involve a large increase in cholesterol as well as the importance of vitamin D and other preventive measures, etc. They are key points in dealing with this population. Most authors come to the conclusion that while knowledge alone is insufficient to significantly alter preventative behavior, habits are unlikely to change without greater understanding. Yet, when attitudes, beliefs, self-efficacy, and a strong call to action are used as mediators, knowledge can have an impact on health-related behaviors [47].

### 7.6. Osteoporosis Management

If a patient is at risk for osteoporosis, nurses should consider which type of individualized intervention is most appropriate for the patient. Therefore, low-risk women should be offered the following lifestyle advice as exercise and adequate daily intake of calcium and vitamin D [12]. In intermediate-risk women, drug treatment is not usually required, but may be considered in addition to lifestyle counseling and adequate daily intake of calcium and vitamin D. When considering treatment, it is important to discuss options with patients and look for risk factors and additional clinical features such as fractures, long-term systemic corticosteroid use, and recurrent fall [12]. Finally, in at-risk women, pharmacological treatment should be considered in addition to lifestyle counseling and adequate calcium and vitamin D intake. Patients with hip fractures and other fragility fractures are considered high-risk patients. After implementing fall prevention strategies and providing lifestyle advice, individuals may be considered candidates for medication. The main goals of osteoporosis treatment are to prevent fractures, maintain or increase BMD, and improve physical function [12]. In this regard, fall prevention strategies should be implemented as about 30% of people in their 60s fall at least once a year and the incidence increases in people in their 80s. Falls have serious consequences for osteoporotic patients. Prevention of falls that can lead to fractures is therefore a priority for older patients. All patients with osteoporosis should be evaluated for fall risk factors. These risk factors include a history of falls, fainting or loss of consciousness, muscle weakness, dizziness or balance problems, visual disturbances, medications such as narcotic analgesics, anticholinergic, and antihypertensives. Environmental factors such as obstacles and poor lighting can also increase the risk of falls [8]. Fall prevention strategies should be discussed with patients, focusing on their homes. Some medical conditions need to be treated, such as dizziness, postural hypotension, poor vision, and inappropriate footwear [20]. Prevention through changes in lifestyle and environment should be the first-line treatment, even before drugs [11].

## 8. Conclusions

Osteoporosis, also known as “silent disease”, is very common among the elderly and is especially important in postmenopausal women, who suffer a sudden loss of bone mass resulting from decreased estrogen production and increased FSH production. Due to an aging population and a longer life expectancy that reaches 85 years today, osteoporosis can be considered a problem for current and upcoming society. Early diagnosis, good prevention work, proper treatment, and fall prevention are the key guidelines to implement. The main actions to consider are: (a) to adopt healthy lifestyle habits (such as a varied and balanced diet) from childhood (which is when the maximum bone mass is formed), with adequate levels of calcium and vitamin D (ensuring a good bone state), and (b) to perform physical exercises (ideally performed outdoors to absorb UV radiation from the sun, the main source of vitamin D). Regarding physical exercise, aquatic exercise should be highlighted as an ideal alternative for this particular population, showing similar benefits to the conventional land-based exercise. It is recommended to perform these exercises at least 60 min 3 times per week, while land-based exercises, such as going for a run, just take 30–60 min 2–3 times per week. Some medications must be taken into account in patients whose situation is more compromised. Bisphosphonates, such as alendronate or risedronate, constitute the first line of treatment. Nevertheless, medication will always complement physical exercise and proper nutrition. Health professionals play an important role in the transmission of information and knowledge of the disease and early diagnosis and prevention. It will depend on their work that the prevalence of osteoporosis continues to increase along with the current aging of the population.

## Figures and Tables

**Table 1:** Number of women with osteoporosis (by 1000) in some European countries (from ref. [10]).

Age Groups	France	UK	Germany	Spain	Italy
50–54	135	127	192	95	128
55–59	200	175	265	126	180
60–64	286	276	328	175	276
65–69	271	308	489	215	335
70–74	364	365	718	270	464
75–79	484	411	672	368	546
80–84	526	417	686	357	558
50–84	2266	2079	3350	1606	2487

**Table 2:** Food calcium and vitamin D content (from ref. [11]).

Food	Ca (mg)	Food	Vitamin D (IU)
Basil	2.113	Cod liver oil	210
Thyme	1.890	Eel	110
Oregano	1.580	Caviar	35
Emmental cheese	1.185	Grilled tuna	25
Semi-skimmed milk powder	1.050	Bonito in oil	24
Zamorano cheese	999	Smoked salmon	19
Laurel	830	Prawns	18
Tetilla cheese	809	Anchovies in oil	11.8
Manchego cheese	765	Eggs	11.4
Cereals, corn, and wheat	453	Cereals, wheat, and rice	8.3
Fresh cheese	338	Sardines	8
Skimmed condensed milk	330	Salmon	8
Sardines in oil	314	Anchovies	8
Eggs	282	Mackerel in oil	4.6
Almonds	252	Muesli	4.2
Milk chocolate	247	Pork liver	2.2
Hazelnuts	226	Chicken liver	1.3
Skimmed Yogurt	183	Beef liver	1.2
Curd	178	Natural, enriched yogurt	1.2
Greek Yogurt	150	Cured cheese	0.9
Chickpeas	143	Skimmed condensed milk	0.8
White beans	126	Croissant or cupcake	0.8
Whole cow’s milk	124	Donut	0.8
Semi-skimmed cow’s milk	114	Serrano ham	0.6

**Table 3:** Daily needs of calcium by age groups (from ref. [11]).

**Babies**	0–6 Months Old	250 mg/day	
	7–12 Months Old	300 mg/day	
**Children and teenagers**	1–3 years old	500 mg/day	
	4–9 years old	800 mg/day	
	10–19 years old	1.300 mg/day	
**Pregnant women**		1.400 mg/day	
**Nursing mothers**		1.500 mg/day	
		Men	Women
**Adults**	20–50 years old	1.000 mg/day	1.200 mg/day
	51–70 years old	1.200 mg/day	1.200 mg/day
**Over 70 years**		1.300 mg/day	

## Data Availability

Not applicable.

## References

[1] Compston J., Cooper A., Cooper C., Gittoes N., Gregson C., Harvey N., Hope S., Kanis J.A., McCloskey E.V., Poole K.E.S. (2017). UK clinical guideline for the prevention and treatment of osteoporosis. Arch. Osteoporos..

[2] Faienza M.F., Ventura A., Marzano F., Cavallo L. (2013). Postmenopausal osteoporosis: The role of immune system cells. Clin. Dev. Immunol..

[3] Curtis E., Litwic A., Cooper C., Dennison E. (2015). Determinants of Muscle and Bone Aging. J. Cell. Physiol..

[4] Vina J., Rodriguez-Manas L., Salvador-Pascual A., Tarazona-Santabalbina F.J., Gomez-Cabrera M.C. (2016). Exercise: The lifelong supplement for healthy ageing and slowing down the onset of frailty. J. Physiol..

[5] Agostini D., Zeppa Donati S., Lucertini F., Annibalini G., Gervasi M., Ferri Marini C., Piccoli G., Stocchi V., Barbieri E., Sestili P. (2018). Muscle and Bone Health in Postmenopausal Women: Role of Protein and Vitamin D Supplementation Combined with Exercise Training. Nutrients.

[6] Svedbom A., Hernlund E., Ivergard M., Compston J., Cooper C., Stenmark J., McCloskey E.V., Jonsson B., Kanis J.A., EU review panel of the IOF (2013). Osteoporosis in the European Union: A compendium of country-specific reports. Arch. Osteoporos..

[7] Curtis E.M., Moon R.J., Harvey N.C., Cooper C. (2017). The impact of fragility fracture and approaches to osteoporosis risk assessment worldwide. Bone.

[8] Gass M., Dawson-Hughes B. (2006). Preventing osteoporosis-related fractures: An overview. Am. J. Med..

[9] Gianoudis J., Bailey C.A., Sanders K.M., Nowson C.A., Hill K., Ebeling P.R., Daly R.M. (2012). Osteo-cise: Strong bones for life: Protocol for a community-based randomised controlled trial of a multi-modal exercise and osteoporosis education program for older adults at risk of falls and fractures. BMC Musculoskelet. Disord..

[10] Kanis J.A., McCloskey E.V., Johansson H., Cooper C., Rizzoli R., Reginster, J.Y. on behalf of the Scientific Advisory Board of the European Society for Clinical and Economic Aspects of Osteoporosis and Osteoarthritis (ESCEO) and the Committee of Scientific Advisors of the International Osteoporosis Foundation (IOF) (2013). European guidance for the diagnosis and management of osteoporosis in postmenopausal women. Osteoporos. Int..

[11] Martin Jimenez J.A., Consuegra Moya B., Martin Jimenez M.T. (2015). Nutritional factors in preventing osteoporosis. Nutr. Hosp..

[12] Tella S.H., Gallagher J.C. (2014). Prevention and treatment of postmenopausal osteoporosis. J. Steroid Biochem. Mol. Biol..

[13] Kamel H.K. (2006). Postmenopausal osteoporosis: Etiology, current diagnostic strategies, and nonprescription interventions. J. Manag. Care Pharm..

[14] Rachner T.D., Khosla S., Hofbauer L.C. (2011). Osteoporosis: Now and the future. Lancet.

[15] Fischer V., Haffner-Luntzer M. (2022). Interaction between bone and immune cells: Implications for postmenopausal osteoporosis. Semin. Cell Dev. Biol..

[16] Bijelic R., Milicevic S., Balaban J. (2017). Risk Factors for Osteoporosis in Postmenopausal Women. Med. Arch..

[17] Osteoporosis—Prevention, diagnosis and treatment (2003). A systematic literature review. SBU conclusions and summary. Lakartidningen.

[18] Sayed S.A., Khaliq A., Mahmood A. (2016). Evaluating The Risk Of Osteoporosis Through Bone Mass Density. J. Ayub Med. Coll. Abbottabad.

[19] Janiszewska M., Firlej E., Dziedzic M., Zolnierczuk-Kieliszek D. (2016). Health beliefs and sense of one’s own efficacy and prophylaxis of osteoporosis in peri- and post-menopausal women. Ann. Agric. Environ. Med..

[20] Maeda S.S., Lazaretti-Castro M. (2014). An overview on the treatment of postmenopausal osteoporosis. Arq. Bras. De Endocrinol. Metabol..

[21] Barrett-Connor E., Siris E.S., Wehren L.E., Miller P.D., Abbott T.A., Berger M.L., Santora A.C., Sherwood L.M. (2005). Osteoporosis and fracture risk in women of different ethnic groups. J. Bone Miner. Res. Off. J. Am. Soc. Bone Miner. Res..

[22] Berard A., Bravo G., Gauthier P. (1997). Meta-analysis of the effectiveness of physical activity for the prevention of bone loss in postmenopausal women. Osteoporos. Int..

[23] Kortebein P., Ferrando A., Lombeida J., Wolfe R., Evans W.J. (2007). Effect of 10 days of bed rest on skeletal muscle in healthy older adults. Jama.

[24] Johnell O., Gullberg B., Kanis J.A., Allander E., Elffors L., Dequeker J., Dilsen G., Gennari C., Lopes Vaz A., Lyritis G. (1995). Risk factors for hip fracture in European women: The MEDOS Study. Mediterranean Osteoporosis Study. J. Bone Miner. Res. Off. J. Am. Soc. Bone Miner. Res..

[25] De Laet C., Kanis J.A., Oden A., Johanson H., Johnell O., Delmas P., Eisman J.A., Kroger H., Fujiwara S., Garnero P. (2005). Body mass index as a predictor of fracture risk: A meta-analysis. Osteoporos. Int..

[26] Kanis J.A., Johansson H., Johnell O., Oden A., De Laet C., Eisman J.A., Pols H., Tenenhouse A. (2005). Alcohol intake as a risk factor for fracture. Osteoporos. Int..

[27] Xu L., McElduff P., D’Este C., Attia J. (2004). Does dietary calcium have a protective effect on bone fractures in women? A meta-analysis of observational studies. Br. J. Nutr..

[28] Darba J., Kaskens L., Perez-Alvarez N., Palacios S., Neyro J.L., Rejas J. (2015). Disability-adjusted-life-years losses in postmenopausal women with osteoporosis: A burden of illness study. BMC Public Health.

[29] Siris E.S., Adler R., Bilezikian J., Bolognese M., Dawson-Hughes B., Favus M.J., Harris S.T., Jan de Beur S.M., Khosla S., Lane N.E. (2014). The clinical diagnosis of osteoporosis: A position statement from the National Bone Health Alliance Working Group. Osteoporos. Int..

[30] Kling J.M., Clarke B.L., Sandhu N.P. (2014). Osteoporosis prevention, screening, and treatment: A review. J. Womens Health.

[31] Simas V., Hing W., Pope R., Climstein M. (2017). Effects of water-based exercise on bone health of middle-aged and older adults: A systematic review and meta-analysis. Open Access J. Sports Med..

[32] Nikander R., Sievanen H., Heinonen A., Daly R.M., Uusi-Rasi K., Kannus P. (2010). Targeted exercise against osteoporosis: A systematic review and meta-analysis for optimising bone strength throughout life. BMC Med..

[33] Carter N.D., Khan K.M., McKay H.A., Petit M.A., Waterman C., Heinonen A., Janssen P.A., Donaldson M.G., Mallinson A., Riddell L. (2002). Community-based exercise program reduces risk factors for falls in 65- to 75-year-old women with osteoporosis: Randomized controlled trial. CMAJ.

[34] Otero M., Esain I., Gonzalez-Suarez A.M., Gil S.M. (2017). The effectiveness of a basic exercise intervention to improve strength and balance in women with osteoporosis. Clin. Interv. Aging.

[35] Liu C., Kuang X., Li K., Guo X., Deng Q., Li D. (2020). Effects of combined calcium and vitamin D supplementation on osteoporosis in postmenopausal women: A systematic review and meta-analysis of randomized controlled trials. Food Funct..

[36] Weaver C.M., Alexander D.D., Boushey C.J., Dawson-Hughes B., Lappe J.M., LeBoff M.S., Liu S., Looker A.C., Wallace T.C., Wang D.D. (2016). Calcium plus vitamin D supplementation and risk of fractures: An updated meta-analysis from the National Osteoporosis Foundation. Osteoporos. Int..

[37] Chevalley T., Brandi M.L., Cashman K.D., Cavalier E., Harvey N.C., Maggi S., Cooper C., Al-Daghri N., Bock O., Bruyere O. (2022). Role of vitamin D supplementation in the management of musculoskeletal diseases: Update from an European Society of Clinical and Economical Aspects of Osteoporosis, Osteoarthritis and Musculoskeletal Diseases (ESCEO) working group. Aging Clin. Exp. Res..

[38] Geyer C. (2017). Postmenopausal Osteoporosis: The Role of Lifestyle in Maintaining Bone Mass and Reducing Fracture Risk. Am. J. Lifestyle Med..

[39] Lim S.Y., Bolster M.B. (2017). Profile of Romosozumab and its potential in the management of osteoporosis. Drug Des. Dev. Ther..

[40] Singh S., Dutta S., Khasbage S., Kumar T., Sachin J., Sharma J., Varthya S.B. (2022). A systematic review and meta-analysis of efficacy and safety of Romosozumab in postmenopausal osteoporosis. Osteoporos. Int..

[41] Balemans W., Ebeling M., Patel N., Van Hul E., Olson P., Dioszegi M., Lacza C., Wuyts W., Van Den Ende J., Willems P. (2001). Increased bone density in sclerosteosis is due to the deficiency of a novel secreted protein (SOST). Hum. Mol. Genet..

[42] Krause C., Korchynskyi O., de Rooij K., Weidauer S.E., de Gorter D.J., van Bezooijen R.L., Hatsell S., Economides A.N., Mueller T.D., Lowik C.W. (2010). Distinct modes of inhibition by sclerostin on bone morphogenetic protein and Wnt signaling pathways. J. Biol. Chem..

[43] Brunkow M.E., Gardner J.C., Van Ness J., Paeper B.W., Kovacevich B.R., Proll S., Skonier J.E., Zhao L., Sabo P.J., Fu Y. (2001). Bone dysplasia sclerosteosis results from loss of the SOST gene product, a novel cystine knot-containing protein. Am. J. Hum. Genet..

[44] Cosman F., Crittenden D.B., Adachi J.D., Binkley N., Czerwinski E., Ferrari S., Hofbauer L.C., Lau E., Lewiecki E.M., Miyauchi A. (2016). Romosozumab Treatment in Postmenopausal Women with Osteoporosis. N. Engl. J. Med..

[45] Shoback D., Rosen C.J., Black D.M., Cheung A.M., Murad M.H., Eastell R. (2020). Pharmacological Management of Osteoporosis in Postmenopausal Women: An Endocrine Society Guideline Update. J. Clin. Endocrinol. Metab..

[46] Alami S., Hervouet L., Poiraudeau S., Briot K., Roux C. (2016). Barriers to Effective Postmenopausal Osteoporosis Treatment: A Qualitative Study of Patients’ and Practitioners’ Views. PLoS ONE.

[47] von Hurst P.R., Wham C.A. (2007). Attitudes and knowledge about osteoporosis risk prevention: A survey of New Zealand women. Public Health Nutr..

